# Successful sutureless repair of multiple left ventricular free wall ruptures due to Takotsubo cardiomyopathy: a case report

**DOI:** 10.1186/s40792-024-01848-3

**Published:** 2024-02-23

**Authors:** Hiroto Yasumura, Koji Tao, Ryo Imada, Yushi Yamashita, Naoki Tateishi, Tamahiro Kinjo

**Affiliations:** Department of Cardiovascular Surgery, National Hospital Organization Kagoshima Medical Center, 8-1, Shiroyamacho, Kagoshima, Kagoshima 892-0853 Japan

**Keywords:** Takotsubo cardiomyopathy, Left ventricular rupture, Sutureless repair

## Abstract

**Background:**

Takotsubo cardiomyopathy (TCM) is a temporary and reversible systolic abnormality of the left ventricular apical area resembling a myocardial infarction. Cardiac rupture due to TCM is a rare but fatal complication. Without cardiac surgery, 94% of patients with left ventricular free wall rupture (LVFWR) due to TCM die. Furthermore, successful surgical cases are rare. We report herein the successful treatment of multiple LVFWRs due to TCM using a sutureless repair.

**Case presentation:**

An 80-year-old man quarreled with his daughter and had a sudden onset of chest pain. He was transferred to our hospital in shock. Electrocardiography showed ST elevation and contrast-enhanced computed tomography revealed a bloody pericardial effusion. Emergent coronary angiography showed no significant stenosis. Cardiac arrest ensued because of cardiac tamponade. Emergent surgery was undertaken and three oozing lacerations on the lateral and inferior walls were noted. A sutureless repair was performed using TachoSil® patches. We also applied Surgicel Nu-Knit® absorbable hemostat with Hydrofit® where TachoSil® failed to completely adhere because of hematoma formation and achieved complete hemostasis. We diagnosed the ruptures due to TCM according to the Mayo criteria. The patient was discharged on postoperative day 71.

**Conclusions:**

A sutureless repair using TachoSil® patches and Surgicel® with Hydrofit® is a minimally invasive and effective method for the treatment of multiple LVFWRs due to TCM.

## Background

Takotsubo cardiomyopathy (TCM), also known as ‘‘broken heart syndrome’’ or ‘‘apical ballooning syndrome’’, is a temporary and reversible systolic abnormality of the left ventricular apical area resembling a myocardial infarction in the absence of coronary artery disease [1]. Cardiac rupture due to TCM is a rare but fatal complication. Without cardiac surgery, 17 of 18 (94%) patients with left ventricular free wall rupture (LVFWR) due to TCM died [2]. Furthermore, successful surgical reports are rare. The cardiac surgeries for LVFWR generally involve suture or sutureless repair. The former technique includes linear closure, infarctectomy, and closure or patch closure, whereas the latter technique includes repair with a collagen patch, such as TachoSil® (CSL Behring, Pennsylvania, USA) [3]. In the current report, we present a patient with multiple LVFWRs due to TCM that were successfully treated using a sutureless repair.

## Case presentation

**Fig. 1:**
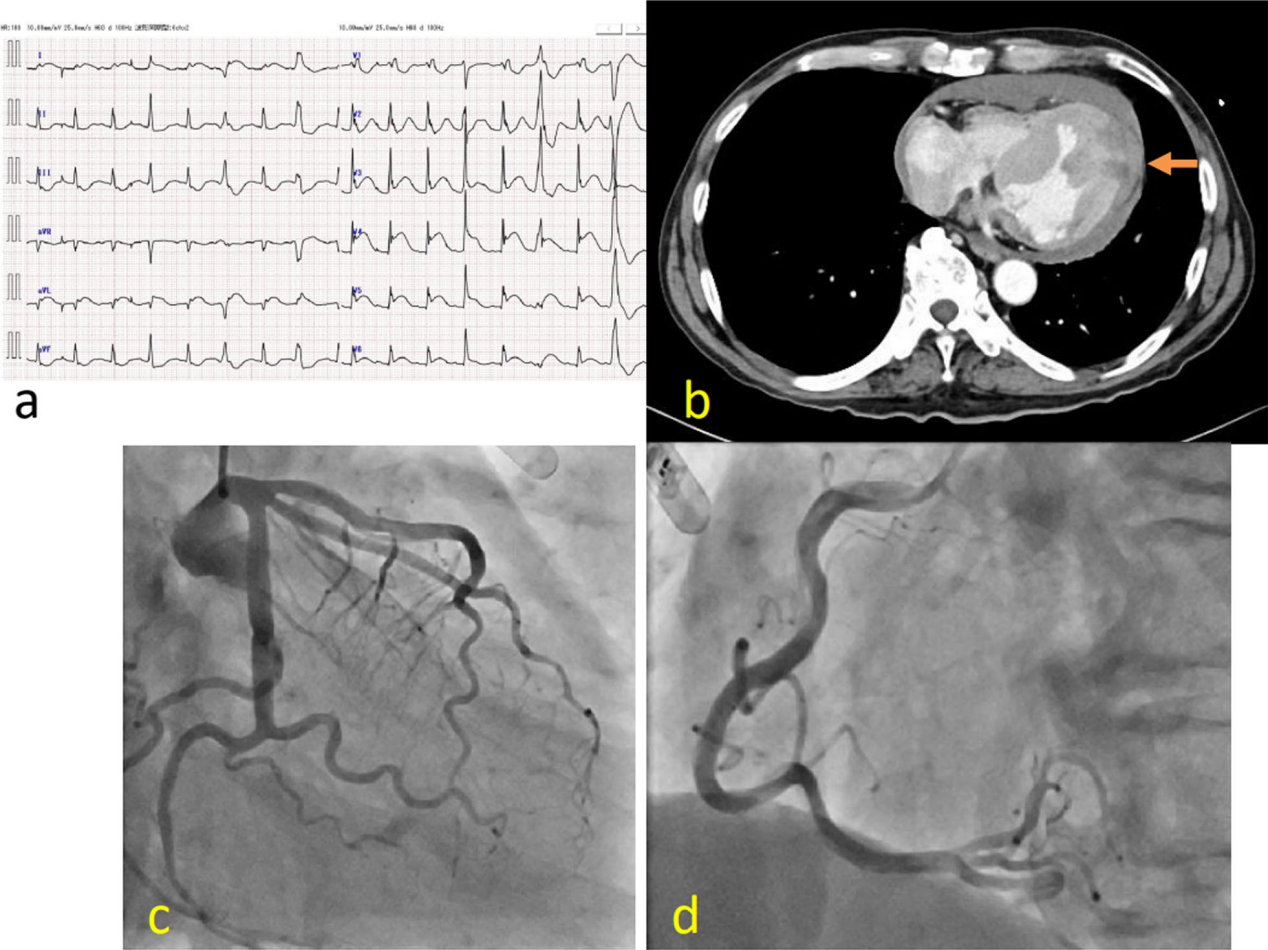
Findings on admission. **a** Electrocardiography showed ST elevation in leads I, aVL, and V3–V6. **b** A contrast-enhanced computed tomography scan revealed a substantial pericardial effusion and a focal low-density area covered with hematoma in the lateral wall (orange arrow). **c**, **d** Emergent coronary angiography showed that there was no significant stenosis. *LAD* left anterior descending artery

**Fig. 2:**
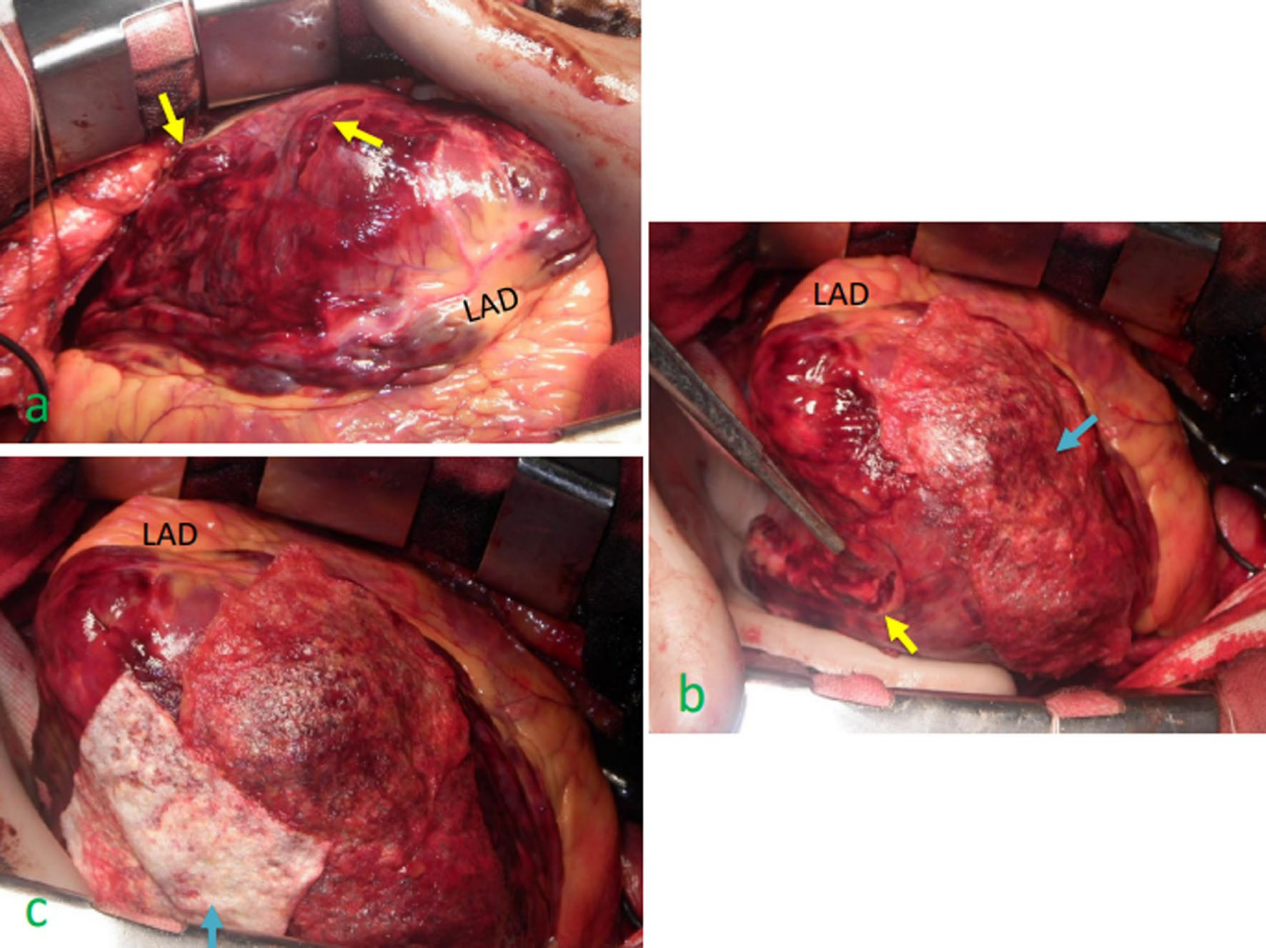
Intraoperative findings. **a** Manual elevation of the heart enabled us to note two oozing lacerations on the lateral wall (yellow arrows). **b** TachoSil® (blue arrow) was applied to the oozing lacerations surrounded by a hematoma on the lateral wall but did not adhere completely because of a wet scaffold. Surgicel Nu-Knit® absorbable hemostat with Hydrofit® successfully adhered to the lacerations. Another laceration was noted on the inferior wall (yellow arrow). **c** TachoSil® (blue arrow) successfully adhered to the laceration on the inferior wall

An 80-year-old man bitterly quarreled with his daughter at night in the summer and had the sudden onset of chest pain while in the shower. He was transferred to our hospital. He had been followed by cardiologists for suspected vasospasm angina, non-sustained ventricular tachycardia, and paroxysmal atrial fibrillation. On physical examination the arterial pressure was 70/59 mmHg and the heart rate was 99 beats per minute. The laboratory data showed an elevated troponin I level (2437 pg/mL [reference value, < 26.2 pg/mL]) and an elevated creatine kinase (CK) level (443 U/L [reference value, 59–248 U/L]). Electrocardiography showed ST elevation in leads I, aVL, and V3–V6 (Fig. 1a). Transthoracic echocardiography (TTE) showed that the ventricles were compressed by a substantial pericardial effusion. Contrast-enhanced computed tomography revealed a bloody pericardial effusion and a focal low-density area in the lateral wall covered with a hematoma (Fig. 1b). Emergent coronary angiography showed that there was no significant stenosis (Fig. 1c, d). He had a pulseless electrical activity arrest due to cardiac tamponade shortly after intra-aortic balloon pumping (IABP) was established. Cardiopulmonary resuscitation and pericardiocentesis restored spontaneous circulation. The hemoglobin level of the > 500 mL drainage fluid was 12 g/dL and did not decrease despite the low blood pressure. Therefore, we performed emergent surgery. Four hours after the diagnosis of LVFWR, a median sternotomy was performed and the femoral artery was simultaneously exposed for cardiopulmonary bypass (CPB). The lateral wall of the left ventricle was covered with a viscous hematoma. After removing the hematoma and manually elevating the heart, we noted three oozing lacerations [two on the lateral wall (Fig. 2a) and one on the inferior wall (Fig. 2b)]. An oozing-type LVFWR was diagnosed and a sutureless repair was performed using TachoSil® fibrin sealant patches (9.5 cm × 4.8 cm and 4.8 cm × 4.8 cm; Fig. 2b, c). Surgicel Nu-Knit® absorbable hemostat (Ethicon, New Jersey, USA) with Hydrofit® (Terumo, Tokyo, Japan) were also applied to the areas where TachoSil® failed to completely adhere because of hematoma formation. In so doing, complete hemostasis was achieved. Hemodynamic parameter was stable with catecholamines decreasing. He was withdrawn from IABP under dobutamine 1 γ(µg/kg/min) and noradrenaline 0.07 γ on postoperative day (POD) 3 to prevent catheter related blood stream infection and begin rehabilitation. He was withdrawn from the respirator on POD 7. The peak CK level was 3729 U/L. TTE showed no pseudoaneurysm on POD 66. There was also no evidence of cardiomyopathy, cerebrovascular disease, or pheochromocytoma, which met the Mayo Clinic diagnostic criteria for TCM (Table 1) [4]. He was discharged from the hospital on POD 71.

**Table 1:** Mayo Clinic criteria for Takotsubo cardiomyopathy

1.	Transient hypokinesis, akinesis, or dyskinesis of the left ventricular mid segments with or without apical involvement; the regional wall motion abnormalities extend beyond a single epicardial vascular distribution; a stressful trigger is often, but not always present
2.	Absence of obstructive coronary disease or angiographic evidence of acute plaque rupture
3.	New electrocardiographic abnormalities (either ST-segment elevation and/or T-wave inversion) or modest elevation in cardiac troponin
4.	Absence of pheochromocytoma or myocarditis

## Discussion

TCM is a temporary, reversible, benign disease, which is often triggered by intense physical or emotional stress. In this case, the patient had no physical stress and a bitter quarrel was considered to trigger TCM. The most serious complication of TCM is LVFWR and rupture of the ventricular septum. The etiology of TCM rupture is unrelated to an acute myocardial infarction (AMI) [5]. Emotional stress increases catecholamine release. Direct catecholamine toxicity on the cardiac muscle cells causes contraction band necrosis, inflammatory cell infiltration, and localized fibrosis [6, 7]. One histopathological case report of LVFWR due to TCM described that myocardial injury involved both ischemic and catecholaminergic etiology [8].

Although TCM rupture occurred within an hour after a quarrel in our case, it generally occurs on days 2–5 of hospitalization [2]. Therefore, LVFWR due to TCM is rare, but should be considered in the differential diagnosis of an unexpected pericardial effusion in the emergency room and on the ward. In our case, there was no significant stenosis of the coronary arteries, thus we did not suspect the presence of TCM and did not perform left ventriculography to confirm TCM.

LVFWR is often accompanied with an AMI and is one of the most severe life-threatening mechanical complications. LVFWR develops in up to 2% of patients with an AMI and accounts for 14–26% of deaths after an AMI [9, 10]. By contrast, LVFWR develops in 1.89% of patients with TCM and accounts for 77% of deaths after TCM [2]. The high mortality rate of LVFWR due to TCM may in part be because AMI is under the watchful eyes of a cardiologist. However, TCM is often caused by stress and other non-cardiovascular diseases, and rupture occurs abruptly under the care of non-cardiologists, thus forcing physicians to withdraw or withhold treatment.

The patient presented herein had an excellent outcome because of prompt diagnosis and appropriate surgical repair. Case reports on the successful surgical repair of LVFWR due to TCM are rare. Indeed, we found only four such surgical cases [11–14] in a PubMed search (Table 2). These case reports lacked detailed descriptions about the rupture type and surgery performed. Our case involved oozing-type LVFWRs and a sutureless repair using collagen patches without CPB. The advantage of a sutureless repair includes the avoidance of CPB and systemic heparinization, and avoidance of suturing a fragile myocardium [3]. Therefore, in cases with multiple oozing-type LVFWRs, sutureless repair is feasible and effective.

**Table 2:** Successful surgical case reports of LVFWR due to TCM

Author(s) and year of publication	Age (years)	Gender (F/M)	LVFWR site	Rupture type	CPB(+)	Surgery	Death(+)
Ishida et al. (2005) [11]	67	F	Unknown	Unknown	Unknown	Unknown	−
Zalewska-Adamiec et al. (2016) [12]	74	F	Apex	Blow out	−	Suture on a double-layered Teflon pad	−
Kudaiberdiew et al. (2017) [13]	63	F	Inferior lateral wall	Unknown	+	Resection of LV pseudoaneurysm and pericardial patch repair	−
Al-Tkrit et al. (2020) [14]	77	F	Unknown	Unknown	−	Large patch repair	+
Present case	80	M	Lateral, inferior wall	Multiple oozing	−	Sutureless repair with TachoSil® patch and Surgicel® with Hydrofit®	−

*LVFWR* left ventricular free wall rupture, *CPB* cardiopulmonary bypass

Sutureless repair may cause postoperative complications, such as re-rupture and pseudoaneurysm. Sutureless repair with TachoSil®/TachoComb® collagen patches (Nycomed, Zurich, Switzerland) is a major emergent treatment option for LVFWR. TachoSil is a medicated sponge coated with human fibrinogen and thrombin and facilitate secondary hemostasis; however, the re-rupture rate after sutureless repair for oozing-type LVFWR due to an AMI was reported to be 12% (4 of 33 cases), occurring on POD 0 in 3 patients and on POD 5 in 1 patient [15]. The re-rupture rate after sutureless repair for a blowout-type LVFWR due to an AMI has been reported to be 0–100% in a limited number of cases [15, 16]. Among patients with re-rupture of an LVFWR, the noteworthy intraoperative findings at the time of initial sutureless surgery with TachoSil®/TachoComb® included wet and unstable scaffold-like oozing of blood from multiple areas of the epicardium, bulging of the infarcted myocardium in the systolic phase, a severely edematous heart, and a large hematoma in the epicardium [15]. In the present case, TachoSil® did not completely adhere to the oozing laceration with a hematoma, but Surgicel® with Hydrofit® sealant successfully adhered to the lacerations. Cases involving the successful use of Hydrofit® sealant for an LVFWR have been recently reported, even for a blowout rupture [17, 18]. Hydrofit® is a viscous diisocyanate prepolymer and has high affinity for wet scaffolds. Water contact initiates the chemical change to form elastomer and adhere rapidly[19]. Surgicel® is an oxidized cellulose polyanhydroglucuronic acid and is able to hold or contain water and blood, which facilitates clot formation. Thus, Hydrofit® is usually reinforced with Surgicel® [17]. The use of Hydrofit® sealant and Surgicel® at the wet scaffold leads to effective hemostasis and provides a dry scaffold for adherence of TachoSil®/TachoComb®. Depending on the condition of the epicardium, a multidisciplinary hemostat approach is imperative for an LVFWR.

## Conclusions

Sutureless repair using a TachoSil® patch and Surgicel® with Hydrofit® is a minimally invasive and effective method for the treatment of multiple LVFWRs due to TCM.

## Data Availability

There are no additional data to disclose.

## Abbreviations

TCM: Takotsubo cardiomyopathy
LVFWR: Left ventricular free wall rupture
IABP: Intra-aortic balloon pumping
POD: Postoperative day
AMI: Acute myocardial infarction
